# The frequencies of different types of nasal septum deviation and their effect on increasing the thickness of maxillary sinus mucosa

**DOI:** 10.15171/joddd.2019.032

**Published:** 2019-10-07

**Authors:** Hamid Taghiloo, Zohreh Halimi

**Affiliations:** ^1^Department of Oral and Maxillofacial Radiology, Faculty of Dentistry, Tabriz University of Medical Sciences, Tabriz, Iran; ^2^Department of Pediatric Dentistry, Faculty of Dentistry, Tabriz University of Medical Sciences, Tabriz, Iran

**Keywords:** CBCT, maxillary sinuses, mucous thickness, septum deviation

## Abstract

***Background.*** Diseases of the paranasal sinuses are very prevalent in East Azerbaijan Province, Iran, which is attributed to various reasons, including environmental and anatomical factors. This study investigated the prevalence of anatomical variations of nasal septum deviation and evaluated the effect of this factor on increasing the mucosal thickness of the sinuses.

***Methods.*** The samples included all the patients referred to Tabriz Faculty of Dentistry, and the frequency of nasal septum deviation in the sample population was evaluated. The samples were re-examined to select the samples with a thickened mucosa of the maxillary sinus. The results were reported using descriptive statistical methods.

***Results.*** Deviation of the nasal septum was seen in 75% of the cases. The results showed that 31.76 % of males and 56.67% of females had an increased maxillary sinus mucosa thickness.

***Conclusion.*** There was a significant relationship between nasal septum deviation and thickening of the maxillary sinus mucosa.

## Introduction

Sinus diseases are categorized as major and prevalent health issues in different communities. Chronic rhinosinusitis (CRS) is a common disease that decreases the quality of life and affects up to 2% of the population worldwide. The gold standard for the diagnosis of chronic rhinosinusitis is the use of CT radiography, which is usually recommended after the failure of medications and before surgery due to the lack of CT scan in medically deprived regions. It is advocated after the failure of medications and before surgery is recommended.^1^ Rhinosinusitis is a major health issue that is associated with an increase in the prevalence of allergic rhinosinusitis all over the world.^2,3^ Reports show that the CT scan can evaluate the nasal cavities, osteomeatal complex, and paranasal sinuses simultaneously. In addition, CT scan is reliable, accurate, and effective in showing the extent of disease and complications related to it.^4^ Natural physiology of the nasal organ relies on the condition of the sinus ostium for proper drainage and ventilation of paranasal sinuses.^5,6^ The ostium is located on the nasolateral area called the osteomeatal complex. The drainage of the osteomeatal complex involves most of the paranasal sinuses. In the case of blockage, it is a pathway of pathogenesis in chronic rhinosinusitis.^7,8^ The human nasal area involves a septum in the midline, separating the right and left cavities. Septal deviation refers to the deviation of the nasal septum in the inner part of the nose, which is categorized as follows:

Type I: A slight deviation in the vertical or horizontal plane that does not stretch through the vertical dimension of the septum.
Type II: Vertical anterior deviation
Type III: Vertical posterior deviation
Type IV: S-shaped septum
Type V: Horizontal spores on one side with or without huge distortion on the opposite side
Type VI: Type V with a deep groove on the concave surface
Type VII: Any combination of Types II to VI.

Nasal septal deviation (NSD) is defined as a deviation of bone or cartilage of the septum (or both) from the midline of the face.^9^ Nasal deviations play a major role in nasal congestion, nasal cosmetic problems, increased nasal airway resistance, and sometimes snoring.^10^ As a result, a comprehensive evaluation of the nasal septum is critical in surgical planning, restoration of function and reconsidering cosmetic issues. Typically, septoplasty is sufficient to treat serious nasal deviations. Approximately 21‒30% of individuals suffer from septal deviations, and severe septal deviation is associated with the prevalence of sinusitis. The role of NSD or concha pneumatization as a potential risk factor for the occurrence of sinusitis has not been properly investigated.^11^ Several radiographic imaging techniques are used to study the maxillary sinus. Panoramic radiography is the most common two-dimensional imaging technique used by most dentists to evaluate the general condition of the orofacial complex.^12,13^ Although panoramic radiography is beneficial for achieving a general view of the orofacial area, it has some inherent limitations, including unequal magnification, and geometric distortion across the whole image layer. These limitations lead to incorrect anatomical and pathological evaluations, as well as unreliable measurement accuracy. Sometimes, the overlapping structures, such as the cervical spines, can lead to misdiagnosis. In addition, important clinical areas might be out of focus (image layers) or might appear faded or even absent. The pathology of the internal wall of the maxillary sinus is often not shown on a panoramic radiograph. Therefore, panoramic radiography cannot be considered as a predictive tool for evaluating the presence of sinus pathology. Obtaining a 3D view using computer tomography (CT) is a more precise method for evaluating the maxillary sinus. Although CT is the choice method for assessing maxillary sinuses, high cost, radiation dose, and access issues have limited its use in general dentistry.^14^ The CBCT technique is a relatively new imaging technology that uses a cone-shaped divergent source, ionizing radiation and a two-dimensional detector called the spinning gantry to record multiple images during a thorough scan around the desired area.^15^ The recent introduction of CBCT (cone-beam computed tomography) has enabled dentists and ENT specialists to understand anatomical abnormalities and pathologic areas within the nasal cavity and paranasal sinuses surrounding them. Inflammation of the mucosa can be easily diagnosed using CT, which has made this method a standard radiographic technique for evaluation of the nasal cavity and paranasal sinuses accurately. Experimental observations of paranasal sinuses using CT (computed tomography) have shown that with the presence of concha bullosa, the nasal septum is deviated to the opposite side convexly and the airway between the concha and the septum is retained. The introduction of CBCT has provided a relatively low-cost and high-resolution alternative for orofacial imaging.^16^ The nasal cavity consists of several structures with specific functions. The nasal septum supports the nasal structures. The medial concha plays a vital role in warming up and moisturizing the respiration airflow.^17^ One of the definitions of concha bullosa includes the theory in which the medial turbinate bullosa is completely pneumatized using this structure. Several reports have mentioned the relationship between concha bullosa and sinusitis, while others have not.^15,18^ Concha bullosa is one of the typical anatomical variations of the medial turbinate. Most patients show symptoms associated with its position and are diagnosed based on headache severity. Many patients diagnosed with concha bullosa simultaneously suffer from the septal deviation and sinusitis as well.^19^ Two types of anatomical variations regarding concha bullosa and deviation of the nasal septum are recognized. Paradoxical concha is a rare situation of nasal obstructive developmental anomaly and refers to the infero-medial position of the medial concha. In this situation, the convex surface of the concha is placed towards the nasal septum and is usually bilateral.^18^ There is a strong relationship between concha bullosa and the deviation of the nasal septum, in which the nasal septum is deviated opposite to concha bullosa. However, the presence of in aerial column between the concha and the septum disregards the role of concha in the expansion of the septal deviation. The presence of sinusitis is more prominent on concha bullosa side; therefore, it seems that the presence of concha bullosa is associated with the formation of sinusitis.^19^ Anatomical variations (nasal septum deviation, concha bullosa and paradoxical medial turbinate) can disrupt the ventilation and drainage of the maxillary sinus, leading to an increase in the risk of mucosal diseases.^12^ Researchers believe that osteomeatal obstruction can prevent ventilation and clearance of mucus from the sinuses.^20-22^

Rudresh S Halawar examined 524 CT images of patients to evaluate septal deviation, reporting that the involvement of sinus groups was more than single sinuses.^28^ Stallman et al^28^ evaluated 998 patients (15 were categorized in both the left and right septal deviation groups since they suffered from bilateral deviation). They concluded that 436 patients had at least one concha bullosa, 227 had one-sided concha bullosa, and 209 suffered from bilateral concha bullosa.^28^ Rudresh S conducted a retrospective study on 883 CBCT scans from September 2005 to June 2008 at the University of Kathryn Dental School, Omaha, NE. All the scans were taken using a 3.0-mm voxel CBCT (Imaging Sciences International) scanner. The scans were reconstructed using the software and evaluated in the sagittal, coronal and frontal views. Gender and age were the only patient-specific variables in this study.^28^ Concha bullosa was defined as the presence of pneumatization of any size in the dorsal, medial and ventral conchae, and the septum deviation was defined as a deviation above 4 mm from the midline. The presence of any radiographic mucosal lesion in the superficial surface of the maxillary sinus was defined as abnormal.^23,24^ Tsai et al^25^ examined 53 CT scans in both the coronal and sagittal views. The presence of nasal deviation was examined at the osteomeatal complex level in both the coronal and sagittal views. According to Orlandi^26^ (2010), the septal deviation was defined as angles >10º at the osteomeatal complex level. The presence of concha bullosa was defined as the pneumatization of the medial turbinate. Twenty-two cases of septal deviation were diagnosed, of which only six showed concha bullosa.

Since the deviation of the septum and concha bullosa are among the most important anatomical variations in the orofacial area, which can lead to multiple issues, including increased thickness of the sinus mucosa and consequently other sinus diseases, understanding the prevalence of these anatomical variations and their effect on the occurrence of sinus disease is of utmost importance. Thus, this study evaluated the prevalence of nasal septal deviation and its relationship with the pathological increase in the mucosal thickness of maxillary sinuses using the CBCT technique.

## Methods

The population in this descriptive-analytical study included all the patients referred to Tabriz Faculty of Dentistry from January 2011 to June 2017. The study aimed to investigate the deviation of the nasal septum and its correlation with the increase in mucosal thickness of maxillary sinuses. The subjects with increased mucosal thickness were classified into two groups of sinusitis and mucositis for detailed examination of mucosal thickening. A local increase in the mucosal thickness was categorized as mucositis and a general increase in the mucosal thickness was categorized as sinusitis. The exclusion criteria consisted of patients with asthma, cystic fibrosis, sinus cysts (mucous retention cyst, etc.), metabolic disorders, malignant diseases, as well as patients with a history of nasal or sinus surgery, traumatic cases and children under 8 years of age.^27^ Patients over 8 years of age, with symptoms of septal deviation and an increase in the mucosal thickness, were selected from all the available CBCT images (coronal section). In the next stage, cases with septal deviation were selected, and the prevalence of septal deviation was assessed in the study population. After that, the samples were evaluated for increased maxillary sinus thickness in the coronal and axial sections. Then, cases of maxillary sinus thickening, comorbid with septal deviation, were selected, and the relationship between the deviation of the septum and an increase in the thickness of the maxillary sinus was evaluated.

The images of the patients were taken using a NewtomVGi Cone Beam CT (Verona, Italy) machine in the Department of Radiology, Faculty of Dentistry, Tabriz University of Medical Sciences. The device delivers a cone-beam x-ray, and has a flat panel detector, 1536×1920 pixels, 127×127-pixel size, 14-bit pixel depth, 360-degree rotation, 4.6-second scan duration, and a maximum voltage of 110 volts. The initial and final reconstruction of the images were carried out with NNT viewer software version 17.2. The radiation specifications of the device were set to automatic.

The data were entered in the NNT Viewer software version 17.2 and then, the images were viewed on a 19" PHILIPS LCD (190B) monitor with a resolution of 1024×1208 pixels and 32 bits in a relatively dark room by two orofacial radiologists. At the end of the study, the agreement between the results obtained by the two observers was measured by the kappa agreement coefficient.

The results of the study were reported using descriptive statistics (frequency of percentages). Chi-squared test was used to evaluate the association between the septal deviation and an increase in the maxillary sinus mucosa thickness. The significance level was considered at P<0.05 and statistical analysis was performed using SPSS 17.

## Results

In this study, the samples consisted of 1,000 cases, of which 100 were eligible to be included in the study (Table 1). According to fi 1, the prevalence of septal deviation was 74.5% in males and 75.5% in females. The prevalence of septal deviation in the whole population was 75%. Chi-squared test showed no significant difference between males and females in the prevalence of nasal septum deviation.

**Table 1:** Frequencies of nasal septum deviation in terms of gender

	**Number of males (%)**	**Number of females (%)**	**Total number (%)**
**Septum deviation**	38 (74.5)	37 (75.51)	75 (25%)
**No septum deviation**	13 (25.5)	12 (24.5)	25 (25%)
**Total**	51 (100)	49 (100)	100 (100%)
**P-value**	0.908		

**Figure 1:**
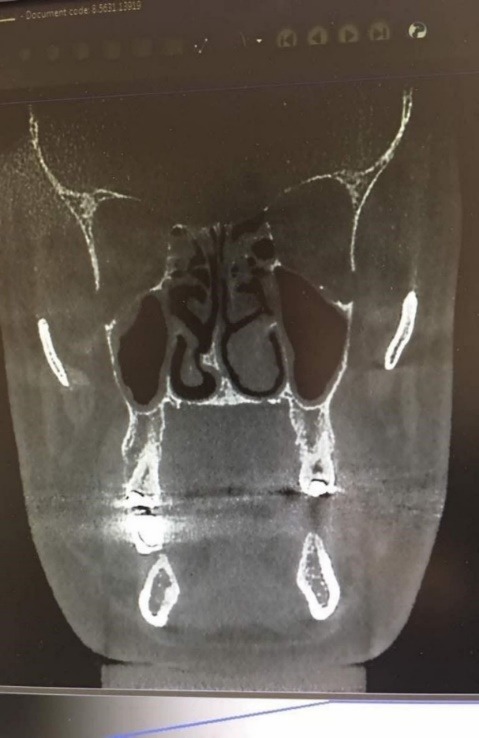
Spore-shaped septal deviation.

Investigation of the frequency of nasal septum deviation in males showed that 33.3% of the deviations were type I, 13.7% were type II, 1.96% were type IV, 17.65% were type V, 1.96% were type VI, and 5.88% were type VII. In females, 26.53% were type I, 14.29% were type II, 2.04% were type III, 8.16% were type IV, 18.37% were type V, 4.08% were type VI, and 2.04% were type VII (Table 2 and Figure 1).

**Table 2:** Frequency of nasal septum deviation in females and males

**Septum type**	**Female**		**Male**	
	**Percentage**	**Frequency**	**Percen ta ge**	**Frequency**
**I**	26.53	13	33.33	17
**II**	14.29	7	13.73	7
**III**	2.04	1		
**IV**	8.16	4	1.96	1
**V**	18.37	9	17.65	9
**VI**	4.08	2	1.96	1
**VII**	2.04	1	5.88	3
**P-value**	0.589			

Chi-squared test did not show any significant difference in the distribution of the frequency of nasal septum deviation between males and females.

The evaluation of the pathological increase in the thickness of the maxillary sinus mucosa showed that the thickness of the mucosa had increased by 31.76 % and 56.67% in males and females, respectively.

**Figure 2:**
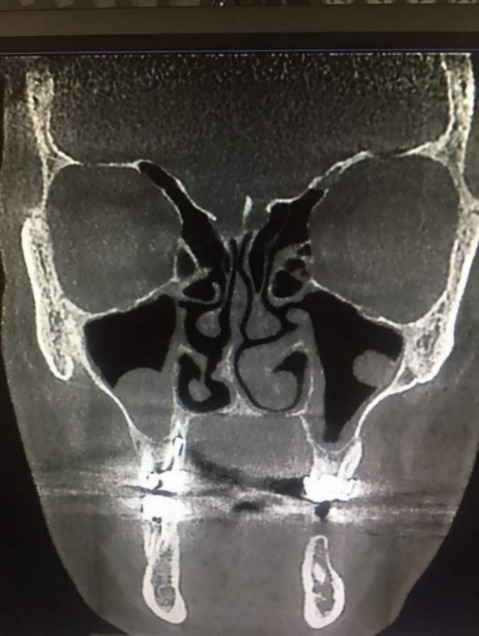
Spore-shaped septal deviation

In males, 23.7% of the increase was normal mucosa, 36.84% was sinusitis, and 39.47% was mucositis. In females, 32.43% of the increase was normal, 18.92% was sinusitis, and 48.65% was mucositis (Table 3 and Figure 2).

**Table 3:** Frequency of pathologic increases in maxillary sinus mucosa thickness in terms of gender

**Septum type**	**Total**		**Female**		**Male**	
	**Percent**	**Frequency**	**Percent**	**Frequency**	**Percent**	**Frequency**
**Normal**	28	21	32.43	12	23.68	9
**Sinusitis**	28	21	18.92	7	36.84	14
**Mucositis**	44	33	48.65	18	39.47	15
**Total**	100	75	100	37	100	38
**P-value**	0.226					

**Figure 3:**
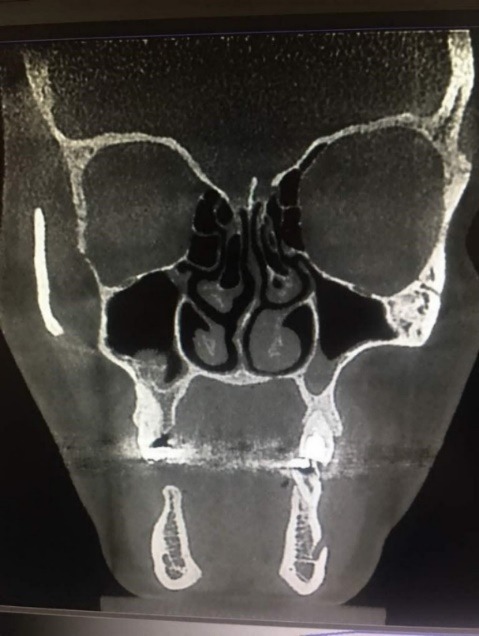
S-shaped septal deviation.

Table 4 shows that in septum types I, VI, II and VII, mucositis was the most common finding. In septum type III, there was one case of normal mucosa. Furthermore, in septum type IV, normal mucosa and sinusitis were more common. In septum type V, sinusitis was the most frequent finding (Figure 3). Chi-squared test did not show any significant difference in the frequency of nasal septum deviation type based on the type of mucosal thickness increase.

**Table 4:** Comparison of the type of nasal septum deviation in terms of the type of mucosal increase

**Septum type**	**Frequency and percentage**	**Type of mucosa increase**			**Total**
		**Normal**	**Sinusitis**	**Mucositis**	
**I**	Frequency	9	7	12	28
	Percentage	32.14	25	42.86	100
**II**	Frequency	1	2	11	14
	Percentage	14.7	14.29	78.57	100
**III**	Frequency	1			1
	Percentage	100			100
**IV**	Frequency	2	2	1	5
	Percentage	40	40	20	100
**V**	Frequency	8	7	5	20
	Percentage	40	35	25	100
**VI**	Frequency		1	2	3
	Percentage		33.33	66.67	100
**VII**	Frequency		2	2	4
	Percentage		50	50	100
	Frequency	21	21	22	75
	Percentage	28	28	29.33	100
**P-value**	0.197				

Table 5 shows that 47.36% of males had concha bullosa, 71% had concha hypertrophy, 18.42% had a closed ostium, and 47.36% had increased thickness of the mucosa of other sinuses. In females, 45.94% had concha bullosa, 54% had concha hypertrophy, 8.1% had closed ostium, and 18.91% had increased thickness in the mucosa of other sinuses.

**Table 5:** Comparison of other findings of the research in terms of gender

	**Male**		**Female**		**Total**	
	**Frequency**	**Percentage**	**Frequency**	**Percentage**	**Frequency**	**Percentage**
**Concha bullosa**	18	47.36	17	45.94	35	46.66
**Concha hypertrophy**	27	71	20	54	47	62.66
**Closed ostium**	7	18.42	3	8.1	10	13.33
**Increased thickness in the mucus of other sinuses**	18	47.36	7	18.91	25	33.33

## Discussion

Rudresh S Halawar study included a number of females twice that of males. In addition, the number of patients with sinusitis was equal to those with normal thickness increase. Generally, this study showed a significant relationship between nasal septum deviation and sinusitis. The involvement of sinuses, such as maxillary, frontal and sphenoid, was more frequent than the involvement of single sinuses. The S-shaped septa and spore-like septa had an equal frequency in that study.^17^ In our study, nasal septum deviation was present in approximately 75% of cases, and there was no difference in the prevalence of septal deviation between males and females. Except for type III, we observed all types of septal deviations in our samples, in which we found that type I and type V were the most frequent. We found no significant difference in the distribution of nasal septum deviation between males and females in our study. The increase in the thickness of maxillary sinus mucosa was 31.76% in males and 56.67% in females. The frequency of mucositis was higher in types I, II, VI and VII nasal septum deviation. In type V septal deviation, the frequency of sinusitis was higher. We observed that the increase in the thickness of other sinuses was present in 33% of cases, which was lower compared to the thickness of maxillary sinus.

The population in the study by Stallman et al^17^ included 60% males and 40% females. Deviation of the nasal septum was present in 65% of cases. In 44% of cases, at least one case of concha bullosa was seen.^17^

However, the number of males and females were approximately equal in our study. In addition, the septal deviation was observed in 75% of our cases. Concha bullosa was seen in 46.6% of cases. A study at the University of Omaha included 883 samples. The samples were examined in the sagittal, coronal and frontal planes. The presence of nasal septum deviation was measured by a 4-mm standard deviation from the midline in our study. The presence of any mucosal lesions in the sinuses was considered abnormal. In this examination, the number of males and females were almost equal as well. No statistically significant relationship was seen between gender and deviation of the nasal septum. Overall, 50% of patients had maxillary sinusitis. In this study, the prevalence of sinusitis was higher in males compared to females, and there was no significant correlation between concha bullosa and sinusitis in patients. This was because 34% of patients were suffering from sinusitis while they did not have symptoms of concha bullosa.^11^

In the present study, the samples were examined in the coronal and axial planes. The criteria for determining septal deviation were based on the Mladina index, which has seven types. The number of males and females were almost equal and similar to the study of the University of Omaha, there was no statistically significant correlation between gender and deviation of the septum. Overall, 31.76% of males and 56.67% of females had increased maxillary sinus mucosa thickness, including mucositis and sinusitis.^11^ Tung-Lung^29^ used the CT scan technique in the coronal and sagittal planes in their study. The criteria to determine nasal septum deviation included any angles of septal deviation >10º in the osteomeatal complex. It was reported that 4% of cases had nasal septum deviations, of which 27% had concha bullosa. Our study included CT scans in the coronal and axial planes. We examined septal deviation according to Mladina, by which we found that 75% of cases had nasal septum deviation, and concha bullosa was seen in 6.46% of the cases.

Therefore, the results obtained from SPSS 17 showed that the significance level was P<0.197, which indicates a significant relationship between nasal septum deviation and thickening of the maxillary sinus mucosa.

## Competing Interests

The authors declare no competing interests with regards to the authorship and/or publication of this article.

## Authors’ Contributions

HT contributed to the concept, experimental studies and manuscript editing. ZH contributed to the design, literature search and data acquisition. Both authors read and approved the final manuscript.

## Acknowledgments

The study protocol was approved by the Ethics Committee in Medical Research of Tabriz University of Medical Sciences (IR.TBZMED.REC.1396.1146).

## Funding

This study was supported by Tabriz University of Medical Sciences, School of Dentistry. Tabriz, Iran [grant no. IR.TBZMED.REC.1396.1146].

## Ethics Approval

The Ethics Committee of Tabriz University of Medical sciences (TUOMS) approved the protocol of this study. All the participants signed informed consent forms (approval no.939).
